# Effectiveness of a Health Educational Program in Enhancing Breast Cancer Knowledge Among Women in Rural Karnataka, South India

**DOI:** 10.7759/cureus.56157

**Published:** 2024-03-14

**Authors:** Mainaz Mainaz, Mohammed Guthigar, Poonam Naik

**Affiliations:** 1 Department of Social Work, Yenepoya (Deemed to be University), Mangaluru, IND; 2 Department of Social Work, Yenepoya Medical College, Mangaluru, IND; 3 Department of Community Medicine, Yenepoya Medical College, Mangaluru, IND

**Keywords:** self-examination, mammogram, clinical breast examination, breast cancer screening, breast cancer knowledge

## Abstract

Introduction and aim: Breast cancer is one of the significant causes of mortality in India, ranking second only to cervical cancer among women. Annually, the country has witnessed the detection of 200,000 new cases, with 60% identified in the early stages. This study aimed to assess the effectiveness of a health education intervention program designed to enhance knowledge about breast cancer among women in rural Karnataka.

Materials and methods: A descriptive study design was employed and a total of 320 women were selected through multi-stage sampling. The educational intervention involved a PowerPoint presentation by the investigator, which was followed by group discussions that culminated with plenary sessions for clarifying the doubts of respondents. At the end of every educational session, pre-designed, pre-tested, and validated questionnaires, comprising a mix of structured and semi-structured questions, were completed by the respondents as part of the post-test.

Results: Among the participants, 44.7% were educated up to the primary level, a majority (64.1%) were employed, and most (90.3%) were married. Additionally, 56.9% reported a monthly income below 3000 Indian rupees (₹), with the majority (86.3%) falling below the poverty line (BPL) category. A statistically significant improvement (p = 0.0001) in knowledge related to breast health, breast self-examination, clinical breast examination, and mammography was observed in the post-intervention phase when compared to the pre-test. 86.2% of the respondents showed an increase in knowledge level about breast health (either from poor to moderate or from moderate to good) and the practice of breast self-examination increased from 4.7% (pre-test) to 60.3% (post-test).

Conclusion: The study demonstrated a significant enhancement in women's knowledge levels after implementing the health education intervention program. These findings underscore the importance of health education strategies in raising awareness of lifestyle diseases, particularly breast cancer, among women.

## Introduction

Breast cancer (BC) remains an arduous global health challenge, persisting as one of the primary contributors to mortality in women despite significant advancements in medical science. In India, the incidence of BC is reported as 162,468 cases with 87,090 deaths occurring yearly [1]. The lack of accessible early detection and treatment options contributes to nearly half of these fatalities, especially in nations with low to moderate economic levels where organized mammography screening remains unattainable. Therefore, urgent public health priorities revolve around early diagnosis and timely treatment [2].

BC, characterized by a malignant tumour originating in the breast cells and predominantly affecting women, possesses the potential to invade healthy tissue and spread beyond its boundaries. Contrary to common misconception, non-communicable diseases such as BC are unavoidable; rather, they represent preventable causes of morbidity, disability, and mortality [3]. Education and awareness campaigns related to BC play a pivotal role in resource-constrained countries, initiating early detection and consequently improving survival rates [4].

It is crucial to educate women on how to detect BC early and seek medical help for any suspicious signs. It is also important to foster a positive attitude towards BC screening using methods such as breast self-examination (BSE), clinical breast examination, and mammography [5]. Since most of the population lives in rural areas, where there are not enough doctors, awareness is not only suitable but necessary [6,7]. Although some studies have examined the awareness of BSE among women, limited literature is available to evaluate the effect of health education interventions on rural women in India.

Hence, this study seeks to address this gap by enhancing the effectiveness of a health education intervention on BC and BSE knowledge among rural women.

Therefore, this study aims to fill this gap by improving the outcome of a health education intervention on BC and BSE knowledge among rural women.

Women in self-help groups (SHGs), acknowledged as agents of empowerment, could, through training, substantially enhance the knowledge of the study population, providing a financially viable and feasible means to promote awareness about BC in rural India [8].

## Materials and methods

Sampling

This study employed multistage sampling, which involved several steps. First, six panchayats were randomly selected from the 59 available in Bantwal Taluk of Dakshina Kannada District, Karnataka, India. Subsequently, the study participants were chosen from three villages using computer-generated random numbers extracted from household survey registers. 

Study design

The research employed a descriptive study design with pre- and post-tests, focusing on women aged 20 to 60 from various SHGs in Bantwal Taluk. Its objective was to sensitize, mobilize, and involve SHG women as catalysts for change to raise awareness among village women about reproductive health, including BC.

Sample size

The study, conducted over one year, included a total of 320 women. Exclusion criteria encompassed women with a history of BC who had previously undergone awareness programs and those who declined to participate. Before conducting the study, ethical permission was taken from the University Ethics Committee. Respondents were provided with a study information sheet and collected signed informed consent forms before administering questionnaires through interview and self-administration. The sample size was calculated using this formula: 

N= (Z_1_^2^-α/2p(1-p))/d^2^,

where d = 5%, Z1-α/2 = 1.96, α = 5%, and p = 71.8%, which gives n = 311, which is rounded off to (N) 320.

Study phases

Phase I (Pre-Interventional Phase)

Data collection tool: The study tool used was a pre-designed, pre-tested, and validated (five experts on the subject), structured and semi-structured questionnaires consisting of items on socio-demographic data (13 questions), knowledge about BC (16 questions), BSE (11 questions), clinical breast examination (four questions), mammography (six questions), attitude (seven questions) rated with five-point Likert scale: strongly agree, agree, neutral, disagree, and strongly disagree. The strongly agree and agree answers to the statement are coded as “positive statements”, the strongly disagree and disagree are coded as “negative statements” and neutral as neutral, and practices regarding BC (seven questions).

Phase II (Interventional Phase)

Health education program: The intervention, named "Breast Health Education - An Early Detection Plan," comprised multiple approaches with interactive sessions, PowerPoint presentations, flipcharts, storytelling, brainstorming, and the distribution of pamphlets in the local language. Three sessions, each lasting 1.5 hours, were conducted for small groups of 20-30 women over the period of six months. Further, the doubts of the respondents were clarified by the investigator while administering post-test questionnaires. The components of the health education model included an introduction to BC, signs and symptoms of BC, diagnostic techniques for BC, the importance of early detection, dispelling myths and presenting facts about BC, demonstration of BSE through a cartoon video, and question-and-answer sessions (Table 1). 

**Table 1:** The health education interactive session plan BC, breast cancer; BSE, breast self-examination; CBE, clinical breast examination.

S. No	Content	Methods	Intervention Details	Duration
1	Introductory session	Slide presentation	Understanding BC: Incidence and factors	10 minutes
2	Signs and symptoms, risk factors	Slide presentation, flipchart, and discussions	All common signs, symptoms, and risk factors of BC	10 minutes
3	Techniques for detection	Slide presentation, flipchart, and discussion	Diagnosis techniques such as BSE, CBE, mammography, sonography, and biopsy	10 minutes
4	Importance of early diagnosis	Slide presentation and discussion	Early diagnosis - benefits and treatments	10 minutes
5	Myths and facts about BC	Discussion	Common myths about BC	10 minutes
6	BSE demonstration	Visual aid and group interaction	Using visual aid and models	20 minutes
7	Question-and-answer session	Discussion	Discussion and doubts-clearing session	20 minutes

Phase III (Post-Intervention Descriptive Phase)

Evaluation: The impact assessment of the health education intervention was conducted six months after the completion of the intervention phase. Respondents were evaluated based on self-reported frequency of BSE, visits to healthcare providers, and mammography screening. Participants with detected abnormalities were referred for further treatment.

Data Analysis

Knowledge, attitude, and practice were quantified as scores, with averages and confidence intervals calculated. The comparison of knowledge scores before and after the intervention utilized the Wilcoxon signed-rank test. Additionally, associations between knowledge levels and demographic variables were examined using chi-square tests. Logistic regression was conducted to explore the impact of socio-demographic variables on knowledge improvement, while McNemar's tests were utilized to compare the nominal variables before and after the intervention.

## Results

The study comprised 320 women, with 44.7% having completed primary education. The majority (64.1%) were employed, and a significant proportion (90.3%) were married. Additionally, 56.9% reported a monthly income below 3000 (₹) rupees, and the majority (86.3%) were classified as part of the category below poverty line (BPL).

At the baseline, 13.8% of the respondents had a moderate level of knowledge of BC and 86.3% had poor knowledge. Education, family income (categorized based on APL [above poverty line] and BPL criteria), and possession of a health card established a connection with knowledge of BC (Table 2). A statistically significant improvement (p < 0.001) in knowledge regarding BC, BSE, clinical breast examination, and mammography was observed after intervention, as determined by the Wilcoxon signed-rank test (Table 3). 

**Table 2:** Association between baseline knowledge and socio-demographic variables using the chi-square test Below 10 = poor, 11-21 = moderate, 22 and above = high knowledge.

Variables		Baseline Knowledge		Total (%)	χ^2^ (p-value)
		Moderate (%)	Poor (%)		
Education	No formal education	4 (6.8)	55 (93.2)	59 (100.0)	9.205 (0.027)
	Primary	15 (10.5)	128 (89.5)	143 (100.0)	
	High school	16 (21.1)	60 (78.9)	76 (100.0)	
	PUC and above	9 (21.4)	33 (78.6)	42 (100.0)	
Family income per month ((₹)	<3000	4 (2.2)	178 (97.8)	182 (100.0)	71.813 (<0.001)
	3001-6000	13 (16.7)	65 (83.3)	78 (100.0)	
	6001-9000	17 (41.5)	24 (58.5)	41 (100.0)	
	9001 & above	10 (52.6)	9 (47.4)	19 (100.0)	
Health card	No	7 (7.1)	91 (92.9)	98 (100.0)	5.2 (0.023)
	Yes	37 (16.7)	185 (83.3)	222 (100.0)	

**Table 3:** Pre- and post-intervention comparison using Wilcoxon signed-rank test Knowledge of breast cancer: 0-11; knowledge of breast self-examination: 0-7; knowledge of clinical breast examination: 0-3; knowledge of mammography: 0-3.

Variable	No. of Questions	Test	Mean	Median	SD	IQR	Test Statistics	p-Value (Wilcoxon signed-rank test)
Knowledge of breast cancer	12	Pre	2.88	2	2.72	4	-14.569	<0.0001
		Post	8.24	9	1.93	3		
Knowledge of breast self-examination	7	Pre	0.59	0	1.41	0	-15.41	<0.0001
		Post	5.54	6	1.2	1		
Knowledge of clinical breast examination	3	Pre	0.84	0	1.01	2	-12.971	<0.0001
		Post	2.13	2	0.56	0		
Knowledge of mammography	3	Pre	0.14	0	0.47	0	-14.828	<0.0001
		Post	1.95	2	0.83	2		
Total	25	Pre	4.46	3	4.66	5.25	-15.284	<0.0001
		Post	17.86	18	3.19	4		

In the pre-test, participants' attitude towards BC, as assessed by the Wilcoxon signed ranking test, was 20.8%, which increased to 26.18% in the post-test (Table 4). Among the 320 participants, 86.2% demonstrated an improvement in knowledge levels after the intervention. Specifically, 18.4% of the participants transitioned from a poor to a high knowledge level, while 66.3% shifted from poor to moderate (Table 5). Furthermore, 60.3% of the participants who initially did not conduct BSE began practicing it after the intervention, representing a notable increase from the pre-test figure of 4.7%. McNemar’s test indicated a statistically significant improvement in the percentage of women conducting BSE before and after the educational program (7.5% vs 65.0%, p < 0.001).

**Table 4:** Pre- and post-intervention comparison of attitude using Wilcoxon signed-rank test The total range of attitude scores is 1-35.

	No of Questions	Mean	Median	SD	IQR	Test Statistics	p-Value
Pre	7	20.80	21	3.999	5	-13.435	<0.001
Post		26.18	26	3.560	5		

**Table 5:** Pre- and post-intervention comparison of total knowledge categories

Baseline Knowledge	Knowledge After Intervention	Frequency	Percentage
Moderate (n = 44, 13.8%)	High	5	1.6
	Moderate	36	11.3
	Poor	3	0.9
Poor (n = 276, 86.3%)	High	59	18.4
	Moderate	212	66.3
	Poor	5	1.6
Total		320	100%

## Discussion

This study aimed to evaluate the impact of a health education intervention program on knowledge, attitudes, and practices related to BC detection and treatment among women. This community-based educational initiative represents the first of its kind among SHGs in the Bantwal Taluk of Karnataka. The findings of the study indicate that following the educational intervention, women showed increased knowledge of BC risk factors and symptoms. However, there was only a modest positive change in attitudes towards BC, its screening procedures, and the inclination to seek medical help.

The study demonstrated a considerable enhancement in BC knowledge during the educational intervention sessions with SHG women. The effectiveness of the intervention appears to be influenced by the teaching method, which involved active engagement in the groups. The educational intervention module, facilitated by the researcher and employing content validated by experts, proved vital. Additionally, providing a demonstration of BSE through a cartoon video and dedicating substantial time to a question-and-answer session was crucial in dispelling myths and enhancing clarity on BC knowledge.

Baseline knowledge of BC and its screening procedures was observed to be low, consistent with similar findings in studies conducted in India, which reported a general lack of awareness about BC and its screening methods [9-11]. The educational program yielded a significant enhancement across all domains of BC knowledge, aligning with other educational interventions conducted in various countries and emphasizing the global impact of such programs [12-14]. Notably, knowledge of risk factors increased, mirroring similar results in a study conducted in Bangladesh. High post-intervention knowledge of BSE, mammography, and clinical breast examination resembled findings from a study in Egypt [15].

The analysis revealed a noticeable rise in knowledge after the intervention among different socio-demographic factors. The intervention effectively improved BC awareness across all educational levels, even among illiterate participants, addressing baseline knowledge disparities [16,17]. Positive shifts in attitude towards BC were noted, consistent with a study conducted in Saudi Arabia. Recognizing that knowledge influences timely and informed decision-making, particularly in early detection and treatment, underscores the critical role of BC-focused educational programs [18].

The study has limitations, including a short follow-up period and the absence of a control group due to the pre- and post-test design. Recommendations include evaluating women over a longer duration and exploring additional aspects such as spousal support, health-seeking behaviour, motivational factors, and attitudes regarding the seriousness of BC. Furthermore, the study acknowledges the limitations of relying on self-reporting for BSE, which has lower sensitivity compared to mammography and clinical examination.

## Conclusions

In conclusion, the results of our study underscore the low level of BC knowledge among rural women in Karnataka, notably concerning symptoms, risk factors, and regular screening procedures. The community-based educational program demonstrated effectiveness in enhancing awareness among the study population. We advocate for healthcare providers to utilize similar educational interventions to mitigate BC-related mortality and advance women's health initiatives.
